# Genotyping panel for assessing response to cancer chemotherapy

**DOI:** 10.1186/1755-8794-1-24

**Published:** 2008-06-11

**Authors:** Zunyan Dai, Audrey C Papp, Danxin Wang, Heather Hampel, Wolfgang Sadee

**Affiliations:** 1 Program in Pharmacogenomics, Department of Pharmacology, Comprehensive Cancer Center, College of Medicine and Public Health, The Ohio State University, 5072 Graves Hall, 333 West 10th Avenue, Columbus, OH 43210-1239, USA; 2 Department of Pathology, College of Medicine and Public Health, The Ohio State University, 680 Ackerman Road, Columbus, Ohio 43202, USA; 3 Division of Human Genetics, College of Medicine and Public Health, The Ohio State University, 2050 Kenny Road, 8th floor tower, Columbus, OH 43221, USA

## Abstract

**Background:**

Variants in numerous genes are thought to affect the success or failure of cancer chemotherapy. Interindividual variability can result from genes involved in drug metabolism and transport, drug targets (receptors, enzymes, etc), and proteins relevant to cell survival (*e.g*., cell cycle, DNA repair, and apoptosis). The purpose of the current study is to establish a flexible, cost-effective, high-throughput genotyping platform for candidate genes involved in chemoresistance and -sensitivity, and treatment outcomes.

**Methods:**

We have adopted SNPlex for genotyping 432 single nucleotide polymorphisms (SNPs) in 160 candidate genes implicated in response to anticancer chemotherapy.

**Results:**

The genotyping panels were applied to 39 patients with chronic lymphocytic leukemia undergoing flavopiridol chemotherapy, and 90 patients with colorectal cancer. 408 SNPs (94%) produced successful genotyping results. Additional genotyping methods were established for polymorphisms undetectable by SNPlex, including multiplexed SNaPshot for *CYP2D6 *SNPs, and PCR amplification with fluorescently labeled primers for the *UGT1A1 *promoter (TA)nTAA repeat polymorphism.

**Conclusion:**

This genotyping panel is useful for supporting clinical anticancer drug trials to identify polymorphisms that contribute to interindividual variability in drug response. Availability of population genetic data across multiple studies has the potential to yield genetic biomarkers for optimizing anticancer therapy.

## Background

Pharmacogenetic studies have shown that polymorphisms in genes related to drug metabolism, transport, and drug targets contribute to interindividual variability in drug efficacy and adverse effects. Hence, pharmacogenetic biomarkers have the potential of optimizing chemotherapy for individual patients [1,2]. This is exemplified with genotyping of thiopurine S-methyltransferase (TPMT), which inactivates thioguanine, to avoid serious toxicity in childhood leukemias [1,3]. Homozygous carriers of defective *TPMT *alleles experience drastically slowed thioguanine inactivation and are at high risk unless the thioguanine dose is reduced more than tenfold. Similarly, deficiency of dihydropyrimidine dehydrogenase (DPYD) activity predisposes to 5-fluorouracil toxicity [4]. Many more examples begin to emerge, with a number of genomic biomarkers listed on the US FDA website for use in guiding drug efficacy and/or safety [5]. In addition, the US FDA has issued "Guidance for Industry Pharmacogenomic Data Submissions" in 2005 for drugs in clinical trials [6]. The purpose is to identify potential biomarkers of interindividual variability in drug response for personalized drug treatment achieving maximum benefit and minimum toxicity. However, the relationship between genotype and phenotype (drug levels, but more importantly, therapy outcome) is confounded by numerous factors, such as age, sex, body weight, nutrition, organ function and comedications, involvement of multiple genes, and population admixture [7]. In this study we have established genotyping panels of relevant candidate genes that could interfere with response to chemotherapy and clinical outcomes; the genotyping panels are flexibly designed so that new candidate genes can be added as needed.

To exploit genetic information in cancer treatment, we must adopt a comprehensive approach, assessing which genes play critical roles in the response to any given drug. For example, irinotecan has become standard in the treatment of intestinal carcinomas. The following genes/proteins could play a role in the response of individual patients: carboxyesterases that activate irinotecan to SN38, CYP3A4 which inactivates irinotecan, UDP-glucuronosyltransferase 1A1 (UGT1A1) which inactivates SN38, and several transporters involved in shuttling irinotecan and SN38 in and out of cells. Among these, (TA)_n_TAA repeats in the promoter region of *UGT1A1 *appear to have a significant impact on irinotecan response and toxicity [8]. This information has been added to the package insert of irinotecan as a warning, and the US FDA has approved a prospective genetic biomarker assay to support individualized dosing [9]. However, given the complexity of the metabolic pathway, the *UGT1A1 *polymorphisms account for only a portion of observed phenotypic variability (e.g., toxicity) [9]. A more comprehensive view of polymorphisms in multiple genes may improve the predictive accuracy of genotype information – even a relatively small increase in predictive power could translate into clinical benefits. In this study we have developed large-scale genotyping methods to provide information on genetic variants of candidate genes involved in drug metabolism and transport.

Drug response is further affected by genes involved in apoptosis, DNA repair, redox cycling, and cell cycle progression. These factors appear to function as main determinants of drug resistance, the principal problem for successful cancer chemotherapy. For example, the DNA-repair enzyme O^6^-methylguanine-DNA methyltransferase (MGMT) is implicated in resistance to alkylating agents [10]. We adopt here a candidate gene approach to determine genetic factors in cancer biology that are likely relevant to an individual's response to chemotherapy. On the other hand, genome-wide SNP analyses are now available using very large-scale array genotyping methods, a trend that might eventually replace candidate gene panels. However, our knowledge of genetic variants in even the most intensely studied candidate genes remains fragmentary, and we expect that long-term, genotyping panels containing only a few strong biomarker genes with complete information on genetic variants will prove valuable clinically.

A second critical factor is the selection of polymorphisms for genotyping within the candidate genes. This involves known functional polymorphisms, polymorphisms of relative frequencies (> 5%) that are likely to affect function (gene regulation, mRNA processing and splicing, translation, and protein functions), and haplotype-tag SNPs providing maximum information on haplotype structures. Numerous Web tools are available to optimize the SNP selection. We summarize here details of the genotyping panels specifically developed for cancer chemotherapy. Similar panels have been proposed elsewhere [11] but the present study extend these panels with further candidate genes to maximize its utility.

Because the polymorphisms/variants differ at the molecular level (SNPs, insertions/deletions, repeats, translocations, LOH, and gene/chromosomal duplications), no single method can readily detect all genotypes. Rather, we first select a versatile method capable of covering a majority of polymorphisms at low cost. The remainder must be completed with a set of varying technologies, at a smaller scale. The aim of this project is to establish a platform for genotyping single nucleotide polymorphisms (SNPs, representing a majority of genetic variants) of genes involved in drug metabolism, transport, and targets, and DNA repair, cell signaling, cell cycle, apoptosis [11-14]. Various high-throughput genotyping platforms are available, each with advantages and disadvantages [15-17]. For example, Affymetrix SNP array is a practical platform for genome-wide genotyping. However, the SNP set is not readily adaptable to include a few newly emerging candidate genes, and the cost for genotyping is relatively high if one wishes to focus on select candidate genes.

In our study, several hundred SNPs need to be genotyped in various numbers of samples. In addition, the SNP set needs to be flexible for different research designs. To establish a flexible, cost-effective, high-throughput genotyping method, we adapted SNPlex genotyping established and systemically validated by Applied Biosystems to have high precision [18,19]. The method can detect 48 SNPs in one single well for each patient sample, adapted here to a 96-well plate format covering more than 400 SNPs for cancer chemotherapy. SNPlex is designed to detect single nucleotide polymorphisms, but not other genetic changes including insertions/deletions and variable number tandem repeat (VNTR) polymorphisms. Additional genotyping strategies, such as multiplexed SNaPshot for *CYP2D6 *and PCR using fluorescently labeled primers to detect the *UGT1A1 *promoter dinucleotide repeat polymorphism, serve as examples of complementary methods. The goal is to generate a common set of genotyping data for cancer treatment trials, thereby, growing the patient and control cohorts for retrospective and prospective analyses. The panel described here can be expanded while new functional polymorphisms are being discovered, and it is suitable for relatively small trials to large cohorts, economically covering up to 1,000 SNPs.

To illustrate potential applications, we show here genotyping results obtained with our SNP panels related to genes involved in cancer biology. For this, we have genotyped a cohort of colorectal cancer patients. In addition, we have applied the drug metabolism and transport gene panels to a Phase I leukemia trial, of which detailed results will be reported elsewhere.

## Methods

### Selection of genes and polymorphisms

The objective was to include genes likely to be involved in therapy outcome. For many of these main candidate genes, genetic studies have already suggested or confirmed functional polymorphisms, but we also include other potential candidate genes/polymorphisms. The main focus of the current study was to include known functional polymorphisms candidate genes based on available literature. The genotyping panels have not been geared primarily to cover all main haplotypes for each gene, but rather to focus on functional SNPs as much as they are known. The purpose therefore is not primarily the discovery of new functional polymorphisms, but rather the assessment of the clinical impact of known ones. We anticipate that in the future we will be able to focus the genotyping panels even more on known functional SNPs, in an effort to develop clinically relevant biomarker panels. The approach takes into consideration that new candidate genes and polymorphisms continue to emerge [20] that need to be flexibly included in the genotyping panels.

We chose candidate SNPs that for the most part have been implicated in cancer biology and chemotherapy in more than one study. For the selected genes, we first surveyed recent reviews for known polymorphisms reported to be related to cancer risk or drug metabolism [12-14], and a lung cancer risk study targeting 250 SNPs in 101 genes [11]. We further searched PubMed for additional polymorphisms associated with cancer risk, revealing genes that are also likely to affect treatment outcome [21]. Lastly, we searched the NCBI dbSNP database for SNPs in the transcribed regions with > 5% minor allele frequency to capture the main haplotypes in genes where only 1–2 SNPs had been selected by the other methods. SNPs from dbSNP were frequent and fully validated by different research projects, such as HapMap project [22] and the NCI SNP500 Cancer project [23]. SNPs in high LD (D'>0.7) with another SNP already in the panel were generally excluded, although in some case we added such SNPs for the assays design, to assure that either one was represented in the panel design. For cytochrome P450 genes, we included the known functional polymorphisms from human allele nomenclature database for cytochrome P450 enzymes [24]. We also searched the UDP-glucuronosyltransferase (UGT) alleles nomenclature database [25] and NAT nomenclature database [26]. We also consulted various drug transporter databases, including the human membrane transporter database [27] and PharmGKB [28].

### SNPlex probe pools and reagents

For the selected SNPs [see Additional file 1], either NCBI SNP reference cluster IDs (rs numbers) or SNP sequences were submitted to Applied Biosystems for the design of SNPlex panels following their proprietary selection algorithms. We separated the genes into different groups: drug metabolism and transport, DNA repair/apoptosis and cell cycle/cell growth/drug targets. DNA sequence surrounding a specific polymorphism must meet specific requirements for probe design, including but not limited to: A. genomic screening; the DNA sequence flanking the target SNP must be unique and not have more than 1 genomic alignment hit with 21 or more contiguous bases to ensure annealing specificity, and there is no second SNP nearby. B. The target sequences should have appropriate features for annealing efficiency. C. Pooling rules: stringent pooling rules are used to determine optimal multiplex composition. SNPlex panels and reagents were synthesized by Applied Biosystems.

### DNA samples

Thirty nine blood DNA samples from chronic lymphocytic leukemia patients were collected by Dr. John Byrd following the institutional review board (IRB) protocol at the Ohio State University for a flavopiridol phase I clinical trials at The Ohio State University Comprehensive Cancer Center. In addition, 90 colorectal cancer samples were chosen from a series of 1262 consecutively accrued patients with colorectal carcinoma diagnosed in the main hospitals of Metropolitan Columbus, whose tumors did not show microsatellite instability, as described previously [29]. Control groups were obtained from previously genotyped cohorts where the same SNPs are accessible (HapMap and other datasets as indicated). The research protocol and consent form were approved by the institutional review board at each participating hospital, and all patients provided written informed consent.

### SNPlex genotyping

**Figure 1:**
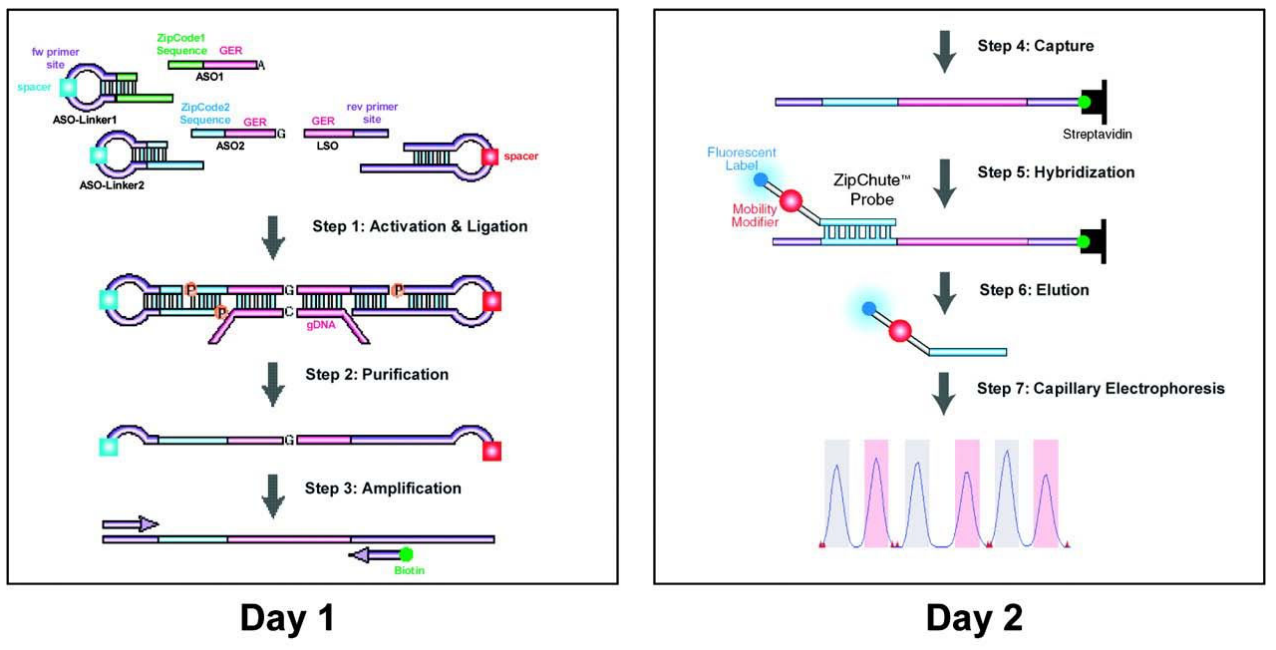
**SNPlex genotyping system assay protocol.** On the first day, the OLA reaction, exonuclease purification, and PCR amplification are performed. On the second day, the amplicons are immobilized on streptavidin-coated microtiter plates. ZipChute probes are hybridized to complementary ZipCode sequences, and non-hybridized ZipChute probes are washed away. The bound ZipChute probes are eluted and analyzed by capillary electrophoresis using Applied Biosystems 3730. Reproduced from Journal of Biomolecular Techniques (Reference [18]) with permission from Andreas R Tobler at Applied Biosystems.

SNPlex genotyping was carried out according to the manufacturer's suggested protocol with slight modifications to accommodate a manual procedure using 96-well plate (90 testing DNAs plus positive and no DNA template controls, and 4 wells for allelic ladders). The multi-step procedure has been previously described (Figure 1) [18,19]. Step 1, for DNA fragmentation, 40 nanogram genomic DNA (2 μl) was fragmented at 99°C for 10 min. Step 2 involved phosphorylation and ligation of allele-specific oligonucleotide (ASO, 2 for each SNP) ligation probes, locus-specific oligonucleotide (LSO, one for each SNP) ligation probes and linkers. SNPlex ASO and LSO ligation probes, universal linkers, dATP and oligonucleotide ligation assay (OLA) master mix containing DNA kinase and ligase were mixed and 3 μl were added to the fragmented DNA. The ligation probes and universal linkers were phosphorylated and then ligated based on the sequence specificity complementary to the genomic DNA template. Step 3, exonuclease digestion served to purify the ligation product. Lambda Exonuclease and Exonuclease I were used to remove unligated probes, universal linkers and genomic DNA. In Step 4, the purified ligation product was PCR amplified using one pair of universal primers, with one primer labeled by biotin. Step 5, the biotinylated PCR products are bound to 96-well streptavidin-coated plate (Sigma). The PCR products were then denatured and the unbounded strands were washed off. Step 6 consisted of hybridization with fluorescence labeled universal ZipChute probe. For 48 SNPs, 96 unique ZipChute probes were hybridized to the complimentary sequence within each allele-specific oligonucleotide (ASO) probes. Each ZipChute probe migrates differently due to a mobility modifier. Step 7, the bound ZipChute probes were eluted and analyzed on Applied Biosystems 3730 DNA Analyzer. Step 8, the elution profiles were analyzed by GeneMapper software (Applied Biosystems) to determine genotypes. With 48 SNPs per run, 48 capillaries in the Applied Biosystems 3730 DNA Analyzer, and up to three injections per hour, the throughput is 5–10,000 SNPs per hour.

### Multiplex SNaPshot genotyping

SNaPshot was performed following a previously published procedure based on single nucleotide primer extension that has been successfully adapted to the Applied Biosystems 3730 DNA Analyzer [30]. A stretch of genomic DNA (50 to 150 base pairs) was amplified by PCR, and the genotype was measured by primer extension using fluorescently labeled terminator nucleotides. Three single nucleotide polymorphisms, rs42427 in the *APC *gene, rs1800392 in *WRN*, and rs2228000 in *XPC*, were multiplexed for the study. Three pairs of PCR primers were amplified simultaneously in 15 μl reactions using 2× ReadyMix™ Taq PCR Reaction Mix with MgCl_2 _(Sigma, St. Louis, MO). For each SNP, 0.15 μl PCR forward and reverse primers (10 μM) were added to the PCR reactions. The amplification was carried out for 30 cycles starting with denaturation at 95°C for 30 s, and then primer annealing at 60°C for 1 min, followed by extension at 72°C for 1 min. The forward and reverse primers were as follows: for rs42427 in *APC*, 5'-CCCTCCAAATGAGTTAGCTGCT-3' and 5'-GCCTTCTGTAGGAATGGTATCTCG-3'; for rs222800 in *XPC*, 5'-GGAGCCATCGTAAGGACCCA-3' and 5'-TGCCTCTTTTACTGCTTGAAGAGC-3'; for SNP rs1800392 in *WRN*, 5'-GGTCCAACAATCATCTACTGTCCTT and 5'-TGATGAATGTCTTTCCTTGTGCTAAA-3'. After PCR amplification, the reactions were treated with Exonuclease I and Bacterial Antarctic Alkaline Phosphatase (New England Biolabs, Beverly, MA). For the primer extension, a gene-specific primer was designed with its 3'-end one base from the SNP position. The forward extension primers were as follows: for rs42427 in *APC*, 5'-TTTTTTTTTTTTTTTTTTCTGGAGAAGGAGTTAGAGGAGG (40 mer); for rs222800 in *XPC*, 5'-TAAGGACCCAAGCTTGCCAG-3' (20 mer), for SNP rs1800392 in *WRN*, 5'-TTTTTTTTCAAGTTACAGGTGAACTTAGGAAACT-3' (34 mer). SNaPshot reagent from Applied Biosystems was used to incorporate a single fluorescently labeled dideoxynucleotide into the 3' end of the primer directed by the DNA template. The primer extension reactions were analyzed using an Applied Biosystems 3730 capillary electrophoresis DNA instrument, and analyzed with GeneMapper 3.0 software (Applied Biosystems), with a throughput of 150 to 750 per hour (if multiplexed to 5 reactions). For *CYP2D6*, the multiplexed SNaPshot was carried out following a previously published protocol with slight modifications [31]. The forward PCR primer (5'-ATGGCAGCTGCCATACAATCCACCTG-3') was redesigned to analyze the promoter SNP rs1080985. The SNaPshot extension primer for rs1080985 was 5'-(T)_58_CCTGGACAACTTGGAAGAACC-3'. A total of 12 polymorphims were analyzed in parallel, by designing extension primers that are separable by capillary electrophoresis.

### Genotyping of *UGT1A1 *promoter (TA)_n_TAA dinucleotide repeat polymorphism

The *UGT1A1 *dinucleotide repeat was genotyped according to previously designed PCR sequences and PCR conditions [32]. The forward primer sequence was 5'-GTCACGTGACACAGTCAAAC-3'. The reverse primer sequence was 5'-TTTGCTCCTGCCAGAGGTT-3' and FAM-labeled. The PCR products were analyzed using an Applied Biosystems 3730 DNA Analyzer.

### Data analysis

Hardy-Weinberg equilibrium for each SNP was analyzed using HelixTree according to the manufacture's manual (Golden Helix, Inc. Bozeman, MT, USA).

## Results

### Genes and polymorphisms selected for genotyping by SNPlex

We have designed cancer genotyping SNPlex panels, selecting genes involved in drug metabolism and transport, DNA repair and apoptosis, cell cycle/cell growth/drug targets. We have selected polymorphisms for genotyping along the following criteria: polymorphisms known to affect enzyme/transporter functions, and SNPs in transcribed genic regions and htSNPs with high abundance obtained from HapMap and other databases. We have selected 560 SNPs for 160 genes, ordered into different categories:

Transporters: ABCA1, ABCA2, ABCA3, ABCA9, ABCA10, MDR1/ABCB1, ABCB4, ABCB11, ABCC1, ABCC2, ABCC3, ABCC4, ABCC5, ABCC6, ABCG2/BCRP, ABCG5, ABCG8, SLC19A1 (RFC) and SLC21A6.

Phase I metabolism enzymes: CYP1A1, 1A2, 1B1, 2A6, 2B6, 2C8, 2C9, 2C18, 2C19, 2D6, CYP2E1, 3A4, 3A5, 17A1, DIA4/NQO1, EPHX1/EH, MPO and SOD2.

Phase II metabolism enzymes: GSTA1 GSTA2, GSTA4, GSTM1, GSTM3, GSTP1, GSTT1, GSTT2, NAT1, NAT2, SULT1A1, SULT1A2, TPMT, COMT, UGT1A1, UGT1A6, UGT1A7, UGT1A9 and UGT2B7.

DNA repair genes: ADPRT/PARP, ADPRTL1, APEX1/APE1, ATM, ATR, BARD1, BLM, BRCA1, BRCA2, CHEK2, ERCC2/XPD, ERCC4/XPF, ERCC5/XPG, FANCD2, LIG1, LIG3, LIG4, MGMT/AGT, MLH1, MPG, MSH2, MSH3, MSH6, MYH/MUTYH, NBS1, NT5E, OGG1, PCNA, PMS2, POLB, RAD23A, RAD51, RAD52, RAD54B, RAD9A, RECQL, WRN, XPA, XPC, XRCC1, XRCC2, XRCC3, XRCC4, XRCC5 and XRCC9/FANCG.

Drug targets, cell signaling, cell cycle and apoptosis related genes: DHFR, DPYD, TYMS, VKORC1, EGFR, ERBB2, FLT1 (VEGFR1), KDR (VEGFR2), FLT4 (VEGFR3), PDGFRA, PDGFRB, KIT, RET, CDA, BAX, CASP3, CASP8, CASP9, CASP10, CCND1, CCNH, CDK7, CDKN1A/p21, CDKN1B/p27, CDKN2A/p16, CDKN2B/p15, GADD45A, IRS2, MDM2, RB1, TERC/hTR, TERT, TP53, TP53BP1, TP53BP2, TP73, APC, NF1, NF2, HPC1, VHL, ECRG1, WT1, MEN1, SMAD2, SMAD4, TNFRSF10A, PTCH and CDH1.

Among the 560 SNPs, 432 SNPs (77%) were successfully designed to be included in the SNPlex panels [see Additional file 1]. The SNPs were divided into several groups so that a subset of the SNPlex panels might be sufficient for a specific research project.

• Drug metabolism and transports: 4 panels, 189 SNPs.

• DNA repair: 3 panels, 148 SNPs.

• Cell cycle/growth/apoptosis: 2 panels, 95 SNPs.

The selection of polymorphisms for this study included some redundancy to account for limitation of the SNPlex approach. Any polymorphisms that could not be included with the SNPlex panels were omitted, or if thought to be critical, targeted by alternative methods. For example, a majority of the SNPs that are not suitable for SNPlex genotyping can be genotyped by multiplexed SNaPshot assay (see multiplexed SNaPshot for *CYP2D6 *in this manuscript as an example). Similarly, small insertions/deletions and repeats can be amplified by PCR and the variants determined by PCR product size difference based on gel electrophoresis or capillary electrophoresis using fluorescent-labeled primers (see *UGT1A1 *promoter dinucleotide repeat polymorphism in this manuscript). Based on the sequence information and literature search, possible alternative methods for detection of these genetic variants are listed in Additional file 1.

### Validation of SNPlex results using SNaPshot

**Figure 2:**
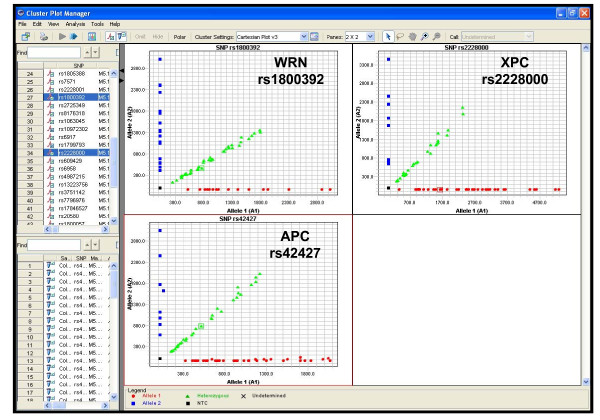
SNPlex genotyping results of three SNPs in colorectal cancer patients were identical to those measured by multiplexed SNaPshot.

We selected SNaPshot (Applied Biosystems), based on single base-pair extension, as a reliable reference genotyping method [33,34]. Three SNPs, rs1800392 in *WRN *gene, rs2228000 in *XPC*, and rs42427 in *APC*, were genotyped using multiplexed SNaPshot. For 74 colorectal cancer samples, the two methods produced identical results for all three SNPs. We monitored the quality of SNPlex genotyping for the three SNPs (Figure 2) to identify the output features indicative of obtaining robust results. The homozygous and heterozygous alleles were clearly separated into different clusters on the Cartesian plots (Figure 2). Similar precision of genotype calls was observed in other projects performed in our laboratory comparing the genotyping results generated from SNPlex, SNaPshot, or TaqMan real-time PCR genotyping (unpublished data). This is consistent with the precision rate that was reported by Applied Biosystems [18].

### Examples of single nucleotide polymorphisms known to be associated with cancer risk or drug treatment response

**Figure 3:**
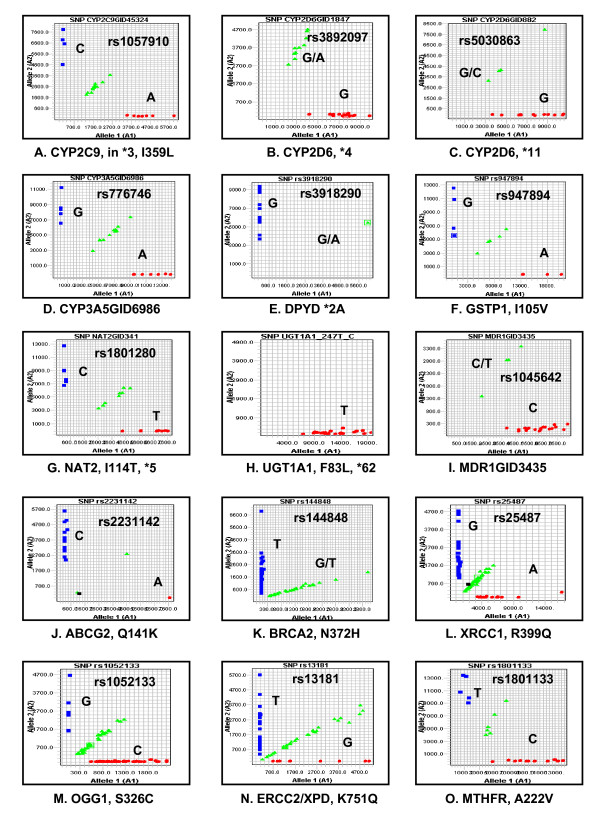
**SNPlex genotyping plots for selected functional SNPs.** Known functional consequences are listed in Table 1 for each SNP.

Examples of the most extensively studied polymorphisms with clinical relevance, such as cancer risk and cancer therapeutic response are summarized in Table 1, which includes 66 SNPs for 24 genes that can be genotyped by our SNPlex panels. We selected 15 SNPs based on their potential significance to cancer treatment response and cancer risk, to illustrate the SNPlex genotyping results (Figure 3). In each case, homozygous and heterozygous genotypes were clearly separated, and therefore, readily assigned.

**Table 1:** Select examples of SNPs with clinical significance.

**Phase I metabolism enzymes**, Allele nomenclature for Cytochrome P450 enzymes [24]:				
**Gene**	**rs#**	**location**	**Function**	**SNPlex**

CYP2C9	rs1799853	*2, R144C	PM 0.25% in Caucasians, life-threatening bleeding after given warfarin	No
	
	rs1057910	*3, I359L		Yes

CYP2C19	rs4244285	*2, 681G>A, exon 5, splicing defect	PM phenotype 2–5% in Caucasians, 18–23% in Asians, > 87% PM in Caucasians is *2 and *3; > 99% PM in Asians has *2 and *3. CYP2C19*2 homozygotes did not respond to antiangiogenic drug thalidomide treatment	No
	
	rs4986893	*3, 17948G>A, exon 4 premature stop		Yes
	
	rs28399504	*4, transcription ablation		Failed
	
		90033C>T, R433W, *5A, *5B	No enzymatic activity	Yes
	
		*7, 19294T>A	Splicing defect, no enzymatic activity	Yes

CYP2D6	rs16947	*2, 2851C>T, R296C	Normal, nucleotide position corrected according to [47]	Yes
	
	rs3892097 or rs1800716	*4, 1847G>A, splicing defect	The CYP2D6 PM is about 5–10% of Caucasians. 99% PM has *3, *4, *5, *6, *7, *8 and *11. *3, *5 and *6 are deletions	Yes
	
	rs28371704	983A>G, H94R	In *4A, *4B, *4F, *4G, *4H and *4J	Failed
	
	rs5030867	*7, 2936A>C, H324P	No enzymatic activity	Yes
	
	rs5030865	*8, 1759G>T	Stop codon, no enzymatic activity	Yes
	
	rs1065852	*10, 100C>T, P34S	Decrease enzymatic activity	Yes
	
	rs5030863	*11, 882G>C	Splicing defect, no enzymatic activity	Yes
	
	rs28371706	*17, 1022C>T, T107I	Decrease enzymatic activity	Yes
	
	rs28371717	*33, 2484G>T, A237S	Normal	Yes
	
		*44, 2951G>C	Splicing defect, no enzymatic activity	Yes

CYP3A4	rs11773597	*1F, m747C>G	Trans-regulation of gene expression is important. Overall, no major pharmacokinetic consequences for the identified *CYP3A4 *SNPs have been observed for the metabolism of anti-cancer drugs [12]	Yes
	
	rs2740574	*1B, m392A>G		Yes
				Yes
	
		*4, 13989A>G,	In AF209389	Yes
	
		*8, 14026G>A	In AF209389, R130Q	Yes

CYP3A5	rs28365083	*2, 27289C>A, T398N		Failed
	
	rs776746	*3, 6986A>G, splicing inclusion	*3 is the most frequent polymorphism (about 90% in Caucasians). Splicing defect, severely decrease of enzymatic activity [12]	Yes
	
	rs28365085	*3d, 31551T>C, I488T		Yes
	
		*5, 12952T>C	Splicing defect	Yes
	
		*8, 3699C>T, R28C	Decreased enzymatic activity	Yes
	
	rs28383479	*9, 19386G>A, A337T	Decreased enzymatic activity	Failed
	
	rs15524	*10, 31611C>T	Decreasde enzymatic activity	Yes

DPYD	rs3918290	splice variant IVS14+1G>A	*2A, Skipping exon 14, ↑ 5FU neurotoxicity [12]	Yes

NQO1	rs1800566	*2, C609T, R187S	*2 and *3 have reduced protein level and enzymatic activity. NQO1 is needed for the activation of mitomycin C, 17AAG (HSP90 inhibitor) and inactivation of benzene-like leukemogenic agents [13]	Yes

	rs4986998	*3, C465T, R139W		Yes

**Phase II metabolism enzymes** NAT allele nomenclature [26]: UGT allele nomenclature [25]:				

**Gene**	**rs#**	**location**	**Function**	**SNPlex**

NAT2	rs1801280	341T>C, I114T, *5A to*5J, *14C and *14F	Alleles with decreased activity include NAT2*5B, NAT2*6A, NAT*7A or B, NAT2*10, NAT2*14A or B, NAT2*17, NAT2*18 and NAT2*19 [12, 14] Low NAT2 activity is related to the increased risk of isoniazid hepatotoxicity	Yes
	
	rs1799929	481C>T, L161L, *5A, *5B, *5F, *5G, *5H, *5I, *6E, *11A, *11B, *12C and *14C		Yes
	
	rs1208	803A>G, K268R,*5B, *5C, *5F, *5G, *5H, *5I, *6C, *12A, *12B, *12C, *12D, *14E and *14F		Yes
	
	rs1041983	282C>T, Y94Y, *13, *5G, *5J, *6A, *6C, *6D, *7B, *12B, *14B, *14D, *14G		Yes
	
	rs1799930	590G>A, R197Q *5E, *5J, *6A, *6B to *6E, *14D		Yes
	
	rs1799931	, 857G>A, G286E *7A, *7B		Yes
	
		499G>A in sequence X14672, E167K, *10		Yes
	
	rs1801279	191G>A, R64Q *14A to *14G,		Yes
	
		434A>C A in sequence X14672, Q145P, *17		Yes
	
		845A>C A in sequence X14672, K282T, *18		Yes
	
	rs1805158	190C>T, R64W, *19		Yes

TPMT	rs1800462	*2, 238G>C	Null genotype associated with hematopoietic thiopurine toxicity, homozygous frequency 1/300 [4]	No
	
	rs1800460	*3A, 460G>A		No
	
	rs1142345	*3C, 719A>G		No

UGT1A1		TA (5–8) TAA	UGT1A1 *28 (7 TAs) associated with increased irinotecan toxicity. Caucasians ~32%	No
	
	rs4148323	211G>A, G71R, *6	Reduced enzymatic activity	Yes
	
	rs34993780	1456T>G, Y486D, *7		Yes
	
	rs35350960	686C>A, P229Q, *27		Yes
	
		247T>C, F83L, *62	Causing Gilbert's syndrome	Yes

GSTT1		Deletion causing null genotype	Null allele has been associated with better or poorer survival in leukemia patients following chemotherapy [12]	No

GSTP1	rs947894	313A>G I105V	Val associated with decreased enzyme activity and increased survival after 5FU/oxaliplatin treatment of colorectal cancer patients [54]	Yes

GSTM1		Deletion causing null genotype	Null allele is associated with increased survival after chemotherapy for multiple cancers [13, 14]	No

SULT1A1	rs9282861	*2, R213H, HaeII	His/His has lower enzymatic activity and is associated with poor survival following tamoxifen therapy [55]	No

**Transporter Genes**				

**Gene**	**rs#**	**location**	**Function**	**SNPlex**

ABCB1	rs1045642	3435C>T	C3435 associated with higher drug transport activity	Yes
	
	rs1128503	1236T>C		Yes
	
	rs2229109	1199G>A		Yes

ABCC2	rs2273697	1249G>A, Val417Ile	1249AA associated with decreased mRNA [56]	Yes

ABCG2	rs2231142	421C>A, Q141K	Minor alleles with lower BRCP expression, enhanced drug sensitivity [12]	Yes
	
	rs2231137	G34 G>A V12M		No
	
		944–949 deletion		No

SLC19A1	rs1051266	80G>A Arg27His	Patients with the 80AA genotype had higher plasma MTX levels, suggesting decreased cellular uptake of MTX	Yes

SLCO1B1/SCL21A6	rs4149056	T521C, Val174Ala, *5	*5 and *15 are associated with decreased transport activity [57]	Yes
	
	rs2306283	Asp130Asn, *15		Yes

**DNA repair genes**				

**Gene**	**rs#**	**location**	**Function**	**SNPlex**

BRCA2	rs144848	N372H	Cancer risk [51]	Yes

OGG1	rs1052133	S326C	Cancer risk [51]	Yes

XRCC1	rs1799782	R194W	Cancer risk [51]	Yes
	
	rs25487	R399Q	Gln399 associated with oxaliplatin/5-FU resistance	Yes
	
	rs25489	R280H		Yes

ERCC2/XPD	rs13181	K751Q	Lys751 associated with improved oxaliplatin/5-FU treatment outcome [52]	Yes

TP53	rs1042522	R72P	Cancer risk	Failed

MGMT	rs12917	262C>T, L84F	Decreased repair of DNA damage [58]	Yes

CHEK2		1100delC	Protein truncation, cancer risk [59]	No

**Drug target, pathway genes**				

**Gene**	**rs#**	**location**	**Function**	**SNPlex**

DHFR	rs5030762	829T>C	SNP 829T>C located in the untranslated region of the DHFR, associated with ↑ of DHFR mRNA, ↓ responsiveness to methotrexate	No

MTHFR	rs1801133	677C>T, A222V	minor allele frequency 24–46%% in Caucasians, T allele is associated with reduced enzyme activity, increased toxicity to methotrexate [13, 53]	Yes
	rs1801131	1298A>C, E429A	Reduced MTHFR enzyme activity [13, 53]	Yes

TYMS		2–9 28 bp repeats in the 5' promoter enhancer	3 repeats ↑ RNA, TSER*3 associated with drug resistance of 5FU and methotrexate	No

CDA	rs2072671	79A>C, K27Q	Minor allele has lower activity to inactivate gemcitabine than the wild-type [60]	Yes
	
		208G>A, A70T	70TT has lower activity to inactive cytidine and ara-C than the wild-type [61]	Yes

**Cell cycle genes**				

CCND1	rs603965	870A>G	Alternative transcript encodes a protein with enhanced cell transformation activity, and modifies caner risk [62]	Yes

### Additional methods to accommodate polymorphisms not suitable for SNPlex

**Figure 4:**
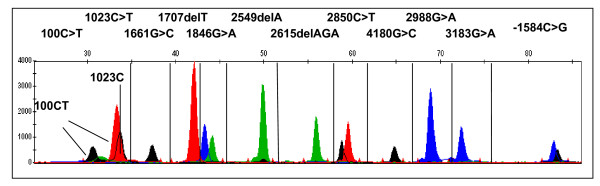
Multiplex SNaPshot genotyping assay for *CYP2D6*.

SNPs excluded from SNPlex panels for genomic sequence and other types of polymorphisms, such as insertions/deletions and variable number tandem repeat (VNTR), can be genotyped by different approaches, including multiplexed SNaPshot, TaqMan PCR, sequencing and SYBR Green melting curve assays [35], to detect all genetic variants of interest for a specific project. Mutiplexed SNaPshot is a primer extension assay with fluorescent terminator dyes, followed by capillary electrophoresis (Applied Biosystems 3730 DNA Aanalyzer). For faster throughput, we multiplex SNaPshot (up to 12 SNPs per reaction) for *CYP2D6*, by using extension primers of varying length (Fig. 4), yielding a throughput of between 150 (single SNP) to 1,800 (multiplex) per hour. Moreover, base pair deletions or insertions can be detected (Fig 4, 1707 del T, 2549 del A, 2615 del AGA).

**Figure 5:**
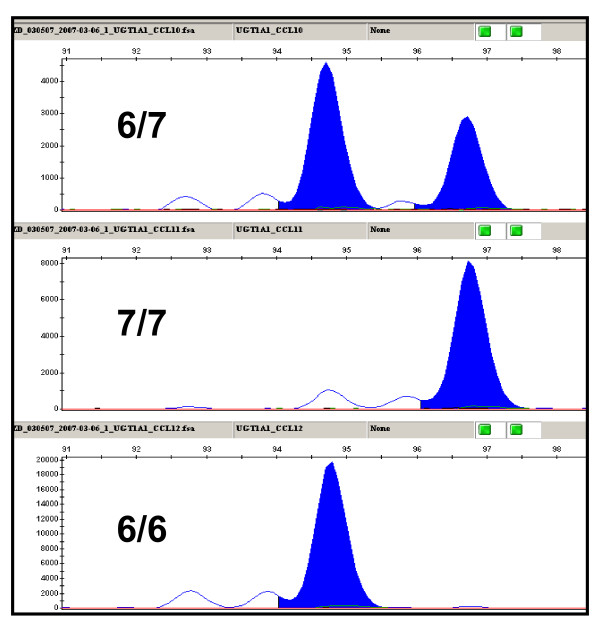
**Genotyping for *UGT1A1 *promoter (TA)nTAA dinucleotide repeat polymorphism.** The three samples are (TA)_6/7_TAA heterozygous, and (TA)_7/7_TAA and (TA)_6/6_TAA homozygous.

The *UGT1A1 *promoter dinucleotide repeat polymorphism, (TA)nTAA, was analyzed by capillary electrophoresis following PCR amplification. The repeats were amplified with unique flanking sequences on each side. The variation of dinucleotide repeat number leads to PCR products of different sizes that can be distinguished by capillary electrophoresis (Applied Biosystems 3730 DNA Analyzer, Figure 5).

### Pilot genotyping study for peripheral blood DNA from leukemia and colorectal cancer patients to evaluate the SNPlex panels

#### A. Genes for drug metabolism and transport in chronic lymphocytic leukemia patients

Flavopiridol, a broad inhibitor of cyclin-dependent kinases, is metabolized by UGT1A9 and UGT1A1, and interacts with a number of transporters, including MRP2, and BCRP (but apparently not with MDR1) [36-40]. Polymorphisms in each of these genes could affect the pharmacokinetic profile, and hence, treatment outcome. Our goals are to genotype and integrate the data with pharmacoanalytical and clinical results. In a pilot project, the 4 drug metabolism and transporter panels (189 SNPs) were applied to 39 DNA samples obtained from peripheral blood of chronic lymphocytic leukemia patients enrolled in flavopiridol clinical trials ongoing at The Ohio State University. To ensure the genotyping quality, the samples with signal intensity for the majority of SNP peaks below 1000 RFU (relative fluorescent units) were eliminated. For the 189 SNPs related to drug metabolizing and transport, 177 (94%) produced successful genotyping information. SNPs with low genotyping quality or that failed entirely in the SNPlex assays are listed in Table 2. The criteria for successful genotyping were based on the information from the manufacture's manual and our validation process (see below). Over 95% (136 out of 146) of the SNPs follow Hardy-Weinberg equilibrium (Chi-squared test, P > 0.01).

**Table 2:** SNPs showing low genotyping quality or failing in the SNPlex analysis.

Panels	DME		Cell cycle	DNA repair
SNPs*	rs6413432 (CYP2E1) rs28371704 (CYP2D6) rs7439366 (UGT2B7) CYP1A2_m730C_T NAT1GID97 CYP2C19GID1	rs4987138 (CYP2D7P1) rs28383479 (CYP3A5) CYP3A5GID27289 CYP1A2GID3534 NAT1GID560 CYP2C19GID80161	rs2066827 (CDKN1B/p27) rs1799939 (RET) rs1042522 (TP53) rs17882155 (TP53)	rs1801321 (RAD51) rs3219489 (MUTYH) rs4986940 (XRCC9) rs3218384 (XRCC2)

*For sequence information, refer to the column in Additional file 1 titled "Unique name in the panel".

#### B. Cancer-related genes in colorectal cancer patients

We genotyped 90 blood samples from Caucasian colorectal cancer patients using the 5 SNPlex panels for polymorphisms in DNA repair and cell cycle/growth/apoptosis. This is a pilot study to identify polymorphisms that contribute to colorectal cancer risk, and possibly treatment outcomes. For the 2 SNPlex panels related to cell cycle/drug target/apoptosis, 91 out of 95 (96%) SNPs were successful. For the three DNA repair panels, 140 out of 144 (97%) were successful (See Table 2 for SNPs showing low genotyping quality or that failed in the SNPlex analyses). Chi-squared test indicates all SNPs follow Hardy-Weinberg equilibrium (P > 0.01).

## Discussion

We have adapted SNPlex as a platform for genotyping 432 SNPs in 160 genes related to the efficacy and toxicity of anticancer chemotherapy, and cancer risk. Stringent quality control criteria were used to attain optimal results. For example, DNA samples with the majority of signal peaks lower than 1000 RFU (relative fluorescence units) were discarded. In addition, DNA quality is a key factor for successful genotyping. Genomic DNA from blood samples and cell lines yielded high success rates. Our pilot studies indicate that 408 SNPs (94%) produced successful genotyping results. This is consistent with a previous study, where 19,779 nonsynonymous SNPs were genotyped by SNPlex in more than 1000 samples for a genome-wide association study [41]. The system allows 48 SNPs/panel to be genotyped simultaneously in each well for one DNA sample in 96-well or 384-well plate format, with a throughput of 5–10,000 SNPs per hour.

The goal is to develop genotyping panels containing polymorphisms shown to be relevant to disease and drug therapy. Therefore, the genotyping platform needs to be flexible to accommodate new findings, while the number of pertinent SNPs remains rather modest at present. In contrast, for discovery of new candidate genes and polymorphisms, very large SNP panels are beginning to be the norm. The SNPlex platform is designed for genotyping assays involving an intermediate number of SNPs (30–500). As each panel is multiplexed to maximally 48 SNPs, multiple panels need to run for larger SNP panel genotyping. As reagent cost is ~$5.00 per run ($0.10/SNP), the method is cost-effective for targeted genotyping of up to 500 to maximally 1000 candidate SNPs. Use of multiple panels permits flexibility in genotyping for specific applications, involving just a few samples or large cohorts. From our experience, for genotyping more than 500–1000 SNPs in any given project, alternative methods such as bead arrays may be more practical because of the increasing number of SNPlex panels needed. However, the optimal method will change rapidly on a yearly basis.

SNPlex is based on DNA ligation; therefore, its specificity is based on the characteristics of DNA sequence. The method can only be used to detect single nucleotide polymorphisms but commonly fails for genotyping repetitive sequences, insertion or deletions, and duplications. In addition, DNA sequence surrounding a specific polymorphism must meet specific criteria for probe design. As a result, the design process will disqualify a number of SNPs for SNPlex genotyping. Approximately 77% of selected SNPs were admissible for SNPlex analysis. Different genotyping strategies, such as multiplexed SNaPshot, TaqMan real-time PCR or sequencing, are complementary for genotyping all types of genetics variants.

An important aspect of this study is the careful selection of candidate genes and SNPs for genotyping. One limitation of the targeted SNP approach is that the panels fall short of covering all functional SNPs. Novel genetic polymorphisms associated with complex diseases, such as cancer, are identified in an increasing pace. For example, results from genome-wide association studies (GWAS) continue to reveal new polymorphisms that suggest the presence of functional variants in candidate genes [42,43]. However, the odds ratios of implicated polymorphisms in these case control studies usually range at or below 1.5, insufficient for inclusion with the intended genotyping panels that are eventually geared towards establishing clinical biomarkers for therapy. Yet, we expect that functional polymorphisms with high odds ratios with respect to specific phenotypes (e.g., treatment outcomes) will emerge from GWAS and its follow-up studies, then to be incorporated into the SNPlex panels. Since the SNPlex platform is flexible and expandable, a small subset of genetic polymorphisms, especially SNPs, could be easily added to the panel established in the current study. In addition, the selection of SNPs was not designed to optimize haplotype tagging – commonly used to survey variation in a gene, and this may represent a limitation of the present panels. Rather, the intent was to genotype a maximum number of SNPs either known or suspected of being functionally relevant – with newly discovered functional variants to be added in additional panels in the future. Moreover, most of the SNPs are in the transcribed regions. We can use these SNPs as markers for analysis of allelic mRNA expression imbalance, a powerful means for discovering regulatory SNPs that alter gene expression and RNA stability. It is estimated that regulatory SNPs are more abundant than nonsynonymous polymorphisms that alter the amino acids [33,34,44,45].

The selected functional SNPs in genes related to drug response and cancer risk are readily detectable using the methods established in the current study (Table 1 and Figure 3). Below, we briefly discuss polymorphisms of clinical significance (see reviews for detailed information [12,13]), to illustrate potential clinical applications of the genotyping panels we have established.

### Phase I metabolizing enzymes

Cytochrome P450's are Phase I drug metabolizing enzymes harboring numerous mutations. For example, the two most important allele variants of *CYP2C9*, CYP2C9*2 and CYP2C9*3, cause a poor metabolizer phenotype associated with adverse warfarin effects [12]. Figure 3A shows the genotyping results with rs1057910 (Fig. 3A) in CYP2C9*3.

CYP2D6 metabolizes many commonly used drugs and is one of the best studied cytochrome P450 enzymes, with numerous variant alleles designated *1 to *61. The incidence of CYP2D6 poor metabolizers, carrying two null alleles, is 5–10% of Caucasians, imparting increased risk of adverse reactions from drugs requiring 2D6 metabolism for elimination. Nearly 99% of poor metabolizers have any two of the following alleles: *3, *4, *5, *6, *7 *8 or *11 [12,46]. Our SNPlex panels include key polymorphisms for alleles *4, *7, *8 and *11. For example, SNP rs3892097 (1847G>A, Fig. 3B), a common SNP in CYP2D6*4A to *4N, causes splicing defect [47], and it accounts for more than 75% of poor metabolizers in Caucasian [46]. CYP2D6*5 is a deletion of the entire functional *CYP2D6 *gene. In alleles *3 and *6, single nucleotide deletions causing CYP2D6 protein reading frame shift are undetectable by SNPlex genotyping. Multiplexed SNaPshot is complementary to SNPlex and can detect alleles *3 and *6 [31]. In addition, 4 SNPs, rs1065852, rs28371706, rs3892097 and rs16947, overlap in SNPlex panels and multiplexed SNaPshot, serving as a quality control. CYP2D6 catalyzes the conversion of tamoxifen to more potent metabolites, and poor CYP2D6 enzymatic activity has been associated with tamoxifen treatment outcome [12].

SNP rs776746 in *CYP3A5 *(6986A>G, CYP3A5*3, Fig. 3D) causes aberrantly spliced mRNA that is unstable, resulting in severely decreases protein level in the liver. The CYP3A5*3 allele frequency is approximately 90% in Caucasians [12].

Dihydropyrimidine dehydrogenase (DPYD) is a rate-limiting phase I metabolizing enzyme for 5-FU inactivation in the liver. SNP rs3918290 (Fig. 3E) is located at an RNA splicing donor site, causing *DPYD *exon 14 skipping (deletion) and leading to inactive enzyme. DPYD deficiency conveys risk for severe, life-threatening 5-FU toxicity [13].

### Phase II metabolizing enzymes

For *GSTP1 *I105V (rs947894, Fig. 3F), Val/Val homozygotes express lower enzyme activity and decreased clearance rate of chemotherapeutic compounds, which leads to an increased survival following 5FU/oxaliplatin treatment of colorectal cancer patients. A better survival was also observed for breast cancer patients following treatment [12].

Polymorphisms affecting acetylator phenotype are common genetic variants for the biotransformation of drugs and carcinogens. *N*-acetyltransferase 2 (NAT2) polymorphisms are among the best studied examples in pharmacogenetics (Table 1 and Fig. 3G, rs1801280). These polymorphisms affect enzyme activity and are associated with drug toxicity and increased risk to develop certain cancers [48].

Uridine diphosphate glucuronosyltransferase 1A1 (UGT1A1) mediates glucocuronidation of bilirubin and anticancer drugs, such as SN38 (active irinotecan metabolite with antitumor activity). The UGT1A1*28 (promoter (TA)_6_TAA to (TA)_7_TAA) is a common genetic variant reducing UGT1A1 activity associated with irinotecan toxicity and hyperbilirubinaemia. Since this is a dinucleotide repeat variation, it is not suitable for detection with SNPlex. Fluorescently labeled PCR was designed to amplify the repeat and flanking DNA region. The repeat number was determined by the PCR product length (Figure 5). The SNPs in UGT1A1*6, *7, *27 and *62 are in the SNPlex panels (Table 1 and Fig. 3H).

### Transporters

ABCB1/Multidrug resistance (MDR1) transporter is an efflux pump. High expression of MDR1 conveys resistance to a number of chemotherapeutic agents, including paclitaxel, doxorubicin and irinotecan [49]. C3435T (rs1045642, Fig. 3I) is a synonymous SNP without amino acid change. However, the T allele has been reported to affect RNA stability [33] and possibly translation [50] and lead to decreased protein expression. Nevertheless, varying results have been reported about the effects of *MDR1 *polymorphisms on pharmacokinetics and pharmacodynamics [12,13]; possibly, the functional polymorphism(s) behave differently in different tissues.

ABCG2 is another extrusion transporter that renders chemoresistance to a variety of anticancer drugs, such as mitoxantrone, methotrexate, doxorubicin and camptothecin-based anticancer drugs [49]. The minor allele of rs2231142 (Q141K, Fig. 3J) is associated with decreased protein expression and results in hypersensitivity to anticancer drugs in caner cell lines [12].

### DNA repair genes

*BRCA2 *N372H (rs144848, Fig. 3K), *XRCC1 *R399Q (rs25487, Fig. 3L), and *OGG1 *S326C (rs1052133, Fig. 3M) are three SNPs in DNA repair genes consistently associated with cancer risk, supported by thirty studies [51]. In addition, the *ERCC2/XPD *variant Lys751Gln (rs13181, Fig. 3N) was associated with the response to treatment with 5-fluorouracil and oxaliplatin in colorectal cancer patients. Lys/Lys homozygotes responded better and had longer survival time [52]. However, contradictory results were observed for cisplatin treatment of non-small cell lung cancer patients [13].

### Drug target/pathway genes

5,10-methylenetrtrahydrofolate reductase (MTHFR), a key enzyme in folate metabolism, catalyzes the conversion of 5,10-methylenetetrahydrofolate to 5-methyltetrahydrofolate, which is involved in DNA and protein synthesis as a methyl donor [1]. SNP rs1801133 (C677T, A222V, Fig. 3O) in *MTHFR *is a functional variant associated with reduced MTHFR enzyme activity in TT homozygotes compared with heterozygots. As a result, the polymorphism increases toxicity to methotrexate [53].

In summary, the selected SNPs have broad applications for cancer research. Furthermore, the SNP panels are not limited to genes involved in cancer treatment outcomes with current drugs in clinical use. Hence, the developed SNPlex panels are not only applicable to studying pharmacogenomics/genetics of novel anticancer compounds under development, but also any drugs for the treatment of other diseases that are metabolized and/or transported by these gene products.

SNPlex has the advantage of being flexible and expandable for different studies, critical for translational research applications, including clinical drug trials. With the implementation of this platform, we have established a pharmacogenomics core with specific application to cancer chemotherapy. We hypothesize that genotyping on a large scale, both with respect to number of polymorphisms and subject populations, will yield valuable information on treatment outcomes. This concept will be applied to Phase I and II clinical trials with novel drugs or drug combinations, in comparison to pharmacokinetic analyses. Availability of population data across all subjects, collected over several years, will support multiple studies and has the potential to reveal novel mechanisms affecting drug response.

## Conclusion

We have established SNPlex as a platform for genotyping more than 400 SNPs in 160 genes related to the efficacy and toxicity of anticancer chemotherapy, and cancer risk. The selected SNPs have broad applications for cancer research to study pharmacogenomics/genetics of current drugs in clinical use and novel anticancer compounds under development. In addition, since the phase I and phase II metabolizing enzymes and transporters are common genes in the absorption and elimination of therapeutic agents for diseases other than cancer, the platform has broad applications for pharmacogenomics studies at large.

## Abbreviations

SNP: single nucleotide polymorphism.

All human gene symbols (names) are approved by HUGO gene nomenclature committee.

## Competing interests

The authors declare that they have no competing interests.

## Authors' contributions

ZD, ACP and DW were involved in the study design, acquisition of data, and interpretation of data, and drafted the manuscript; HH collected and classified all colorectal cancer samples, and assisted in drafting the manuscript; WS conceived the study, was responsible for its design and coordination, helped in the evaluation of the results and revised the manuscript critically for important intellectual content. All authors read and approved the final manuscript.

## Pre-publication history

The pre-publication history for this paper can be accessed here:



## Supplementary Material

Additional file 1 Selected genetic polymorphisms in 160 genes for cancer pharmacogenomics. This table includes all selected genes, genetic polymorphisms, sequences or rs numbers for selected SNPs. Click here for file
